# IUC24414-80 Optimizing sunitinib therapy in mRCC through pharmacological counseling and early TDM integration

**DOI:** 10.1093/oncolo/oyaf248.040

**Published:** 2025-08-29

**Authors:** G Bortolus, E Cecchin, S Gagno, B Posocco, A Dri, S Santarossa, G Toffoli, F Puglisi, M Spina, L Fratino

**Affiliations:** Department of Medicine DMED, University of Udine, Udine 33100, Italy; Experimental and Clinical Pharmacology Unit, Centro di Riferimento, Oncologico di Aviano CRO, IRCCS, Aviano 33081, Italy; Experimental and Clinical Pharmacology Unit, Centro di Riferimento, Oncologico di Aviano CRO, IRCCS, Aviano 33081, Italy; Experimental and Clinical Pharmacology Unit, Centro di Riferimento, Oncologico di Aviano CRO, IRCCS, Aviano 33081, Italy; Department of Medical Oncology, Centro di Riferimento, Oncologico di Aviano CRO, IRCCS, Aviano 33081, Italy; Department of Medical Oncology, Centro di Riferimento, Oncologico di Aviano CRO, IRCCS, Aviano 33081, Italy; Experimental and Clinical Pharmacology Unit, Centro di Riferimento, Oncologico di Aviano CRO, IRCCS, Aviano 33081, Italy; Department of Medicine DMED, University of Udine, Udine 33100, Italy; Department of Medical Oncology, Centro di Riferimento, Oncologico di Aviano CRO, IRCCS, Aviano 33081, Italy; Department of Medical Oncology, Centro di Riferimento, Oncologico di Aviano CRO, IRCCS, Aviano 33081, Italy

## Abstract

**Background:**

Sunitinib (SUN), a standard treatment for metastatic renal cell carcinoma (mRCC), demonstrates considerable interpatient variability in drug exposure and therapeutic response. Fixed dosing may lead to suboptimal drug levels, resulting in under- or over-exposure. The integration of therapeutic drug monitoring (TDM), pharmacogenetic (PGx) analysis, and drug–drug interaction (DDI) assessment may help optimize treatment outcomes. This study aimed to evaluate the feasibility and clinical utility of enhanced pharmacological counseling in the management of SUN therapy for mRCC.

**Methods:**

Blood samples were collected at steady-state to measure trough plasma concentrations (Cmin) in patients enrolled in the CRO-2022-14 trial. SUN plasma levels were quantified using a validated LC-MS/MS method. Target Cmin ranges for efficacy and safety were defined as 37.5-60/75 ng/mL for continuous dosing and 50-80/87.5 ng/mL for intermittent dosing. Genetic polymorphisms related to SUN metabolism and transport were analyzed, and DDIs were assessed using the UpToDate Lexi-Drugs tool. Oncologists received integrated pharmacological reports based on the collected data.

**Results:**

Among the 14 enrolled patients, 12 were receiving reduced doses due to toxicity concerns. Six patients (67%) treated with 25, 37.5, or 50 mg/day achieved target Cmin levels (53-79 ng/mL). One female patient exhibited Cmin >80 ng/mL despite a dose reduction to 25 mg/day, correlating with recurrent grade 2/3 toxicities and suggesting the need for further dose adjustment. Conversely, 2 male patients receiving 25 mg/day showed subtherapeutic Cmin levels (<40 ng/mL) and reported absent or mild toxicity, supporting potential dose escalation. Female patients (*n* = 2) had higher mean Cmin levels (81 ng/mL) compared to males (*n* = 12, 65 ng/mL), though the small sample size limits definitive conclusions. PGx analysis was preliminary, and no clinically relevant DDIs were identified.

**Conclusions:**

In this cohort, toxicity-driven dose modifications enabled optimal SUN exposure in 67% of patients. Early TDM integration appears promising for refining individualized dosing strategies, improving tolerability, adherence, and therapeutic efficacy. Observed sex-based differences in SUN plasma exposure warrant further investigation. Pharmacological counseling proved feasible and clinically impactful, supporting its implementation in routine mRCC management.

